# Characterization of bronchus-associated lymphoid tissue induced by co-exposure to Asian sand dust and ovalbumin: a study using 3D serial section imaging

**DOI:** 10.3389/fimmu.2025.1578255

**Published:** 2025-08-04

**Authors:** Yoshikazu Mikami, Manabu Hayatsu, Etsushi Kuroda, Hiromasa Tsuda, Taku Toriumi, Yusuke Mizutani, Takamichi Ichinose, Akiko Honda, Hirohisa Takano

**Affiliations:** ^1^ Division of Microscopic Anatomy, Graduate School of Medical and Dental Sciences, Niigata University, Niigata, Japan; ^2^ Department of Immunology, Hyogo College of Medicine, Nishinomiya, Japan; ^3^ Department of Biochemistry, Nihon University School of Dentistry, Tokyo, Japan; ^4^ Department of Anatomy, The Nippon Dental University School of Life Dentistry at Niigata, Niigata, Japan; ^5^ Office of Institutional Research, Hokkaido University, Hokkaido, Japan; ^6^ Graduate School of Global Environmental Studies, Kyoto University, Kyoto, Japan; ^7^ Graduate School of Engineering, Kyoto University, Kyoto, Japan; ^8^ Institute for International Academic Research, Kyoto University of Advanced Science, Kyoto, Japan; ^9^ Research Institute for Coexistence and Health Science, Kyoto University of Advanced Science (KUAS-RICH), Kyoto, Japan

**Keywords:** iBALT, Asian sand dust, allergen, 3D imaging, T_fh_ cells

## Abstract

**Introduction:**

Inducible bronchus-associated lymphoid tissue (iBALT) develops with different morphologies and functions depending on the type of antigen, in which various cytokines, such as interleukin (IL)-1 and IL-17, and the cells producing them, such as T helper 17 (Th17) and T follicular helper (T_fh_) cells, play an important role. We recently observed that numerous inflammatory cells, mainly B cell like-cells forming peribronchial clusters, accumulate in the lungs of mice exposed to Asian sand dust (ASD), suggesting that ASD induced iBALT development. However, whether ASD induced iBALT formation, much less the mechanism by which ASD promotes iBALT formation, remains unknown.

**Methods:**

B cell clusters were analyzed using the next generation serial section-three-dimensional (nSS3D) imaging method, in which we attempted to introduce batch image acquisition using a high-resolution slide scanner and AI-based image registration and target extraction. Furthermore, the mechanism underlying ASD-induced B-cell cluster formation was examined using CD4-Cre Bcl6^f/f^ mice lacking T_fh_ cells.

**Results:**

ASD induced B-cell cluster formation in mouse lung tissue, which was enhanced by allergen (Ovalbumin: OVA) exposure. Furthermore, the nSS3D images revealed that a part of the B cell clusters induced by OVA+ASD but not others exhibited common histological features of previously reported iBALTs. Moreover, OVA+ASD exposure failed to induce all of the B cell cluster formation including iBALTs in CD4-Cre Bcl6^f/f^ mice.

**Conclusion:**

B cell clusters including iBALTs are induced by ASD; this process is enhanced by OVA, in which T_fh_ cells were suggested to play important roles. Characterization of the OVA+ASD-induced B cell clusters proved that the SS3D technique is useful for the analysis of mouse disease models. The results also emphasize the need for medical countermeasures for patients with allergic diseases living in areas with ASD contamination.

## Introduction

Asian sand dust (ASD) is a significant public health concern in East Asia, where large quantities of mineral dust are transported from arid regions of China and Mongolia to densely populated areas such as Japan and Korea. Several epidemiological studies have shown that ASD exposure is associated with increased hospital admissions for respiratory diseases, including asthma exacerbations (1, 2). Furthermore, ASD particles often carry microbial and chemical components, which may enhance their immunogenic and inflammatory potential (3–5).

Bronchus-associated lymphoid tissue (BALT) is a submucosal lymphoid follicle consisting mainly of lymphocytes, but also macrophages and dendritic cells, and an important lung mucosal immune system component, which (6, 7) distributes antigen-specific antibody-producing cells into the lungs (8, 9). BALT is present in normal lung tissue in some animal species, such as rats and rabbits (traditional BALT), whereas in others, including humans and mice, it is rare under normal physiological conditions but often develops due to viral or bacterial infections, allergen exposure, or autoimmune diseases. Traditional BALT is a secondary lymphoid organ; however, the latter is an organized tertiary lymphoid organ that is not pre-programmed, and is known as inducible BALT (iBALT). Both traditional BALT and iBALT are involved in pathological conditions (10, 11). As BALT is involved in adaptive immune responses, it contributes to protective humoral and cellular immunity against pathogens such as bacteria, viruses, and environmental air pollutants. However, in the context of autoimmune diseases, it may potentially aggravate tissue damage. Therefore, understanding and controlling the pulmonary immune mechanisms involving traditional BALT and iBALT is essential for immune system research. We recently found that exposure to ASD led to the formation of immune cell clusters, primarily composed of B-cell like cells, around bronchi in the lungs, suggesting the development of iBALT (12). Moreover, several epidemiological studies suggest that exposure to environmental particulates, such as ASD, can exacerbate lung allergic diseases, such as asthma (1, 2). Additionally, our previous studies have shown that ASD worsens inflammation through the infiltration of lymphocytes and macrophages in the lungs of an asthma mouse model induced by allergen (ovalbumin: OVA) exposure (3, 4). These findings suggest that ASD is an exacerbation factor of allergic respiratory diseases and that iBALT formation may be involved in the mechanism underling them. Meanwhile, it had been reported that at least three common events initiate iBALT formation: (i) activation of innate pattern recognition receptors that recognize pathogen-associated molecular patterns; (ii) production of inflammatory cytokines (such as TNF-α or lymphotoxin) and lymphoid chemokines by hematopoietic or stromal cells; and (iii) activation/maturation of antigen-presenting cells and the development of high endothelial venules (HEVs) that promote lymphocyte homing (13). However, iBALT develops, at least in part, with different morphologies and functions depending on the pathogenic conditions, where various cytokines, such as interleukin (IL)-1 and IL-17, and the cells producing them, such as T helper 17 (Th17) and T follicular helper (Tfh) cells, play an important role (10, 11, 13, 14). However, whether ASD induced iBALT formation, and the molecules and cells responsible for the ASD-induced iBALT formation and exacerbation of pulmonary allergic disease were unclear. Therefore, the first aim of this study was to determine whether exposure to ASD induces the formation of iBALT. In addition, we investigated how this process is modulated in a murine model of allergic airway disease induced by ovalbumin (OVA). To this end, we histologically characterized ASD+OVA-induced B cell-like cell clusters to determine whether they represent iBALT, and attempted to identify the cells that play a critical role in ASD+OVA-induced iBALT formation using CD4-Cre Bcl6^f/f^ mice, which lack T follicular helper (T_fh_) cells (14).

Three-dimensional observation is important for the pathological analysis of complex biological tissues such as the lungs. Recently, the combination of tissue transparency technology and light-sheet fluorescence microscopy has attracted significant attention (15, 16). Although this technique is extremely useful, it can be difficult to apply when large amounts of foreign matter, such as environmental particles, are present in tissues, because they block fluorescence. On the other hand, the traditional method of constructing 3D images by integrating serial section images (SS3D) is not practical because of the enormous amount of time and effort required. We have attempted to address these problems by introducing batch image acquisition using a high-resolution slide scanner and AI-based image registration and target segmentation to the SS3D method (next generation SS3D: nSS3D). This technology enables analyses to be completed in as little as one week to analyze one organ in a mouse, whereas previous methods using SS3D, which were performed manually by humans, required several months. We have recently reported the study applied this technology to analyze liver fibrosis in a mouse model of hepatitis, showing that nSS3D could detect the degree of fibrosis more clearly than conventional observation of liver tissue sections stained with Azan. Furthermore, we successfully achieved quantitative analysis of fibrotic tissue (17). However, it remains to be verified whether this technique is equally effective for the analysis of other organs. In particular, the performance of AI learning is expected to vary depending on the target tissue and structural characteristics. Therefore, as the second task of this study, we assessed the applicability of nSS3D technology for the analysis of mouse lungs by examining the induction of iBALT formation by ASD.

## Materials and methods

### Animal experiments

**Figure 1:**
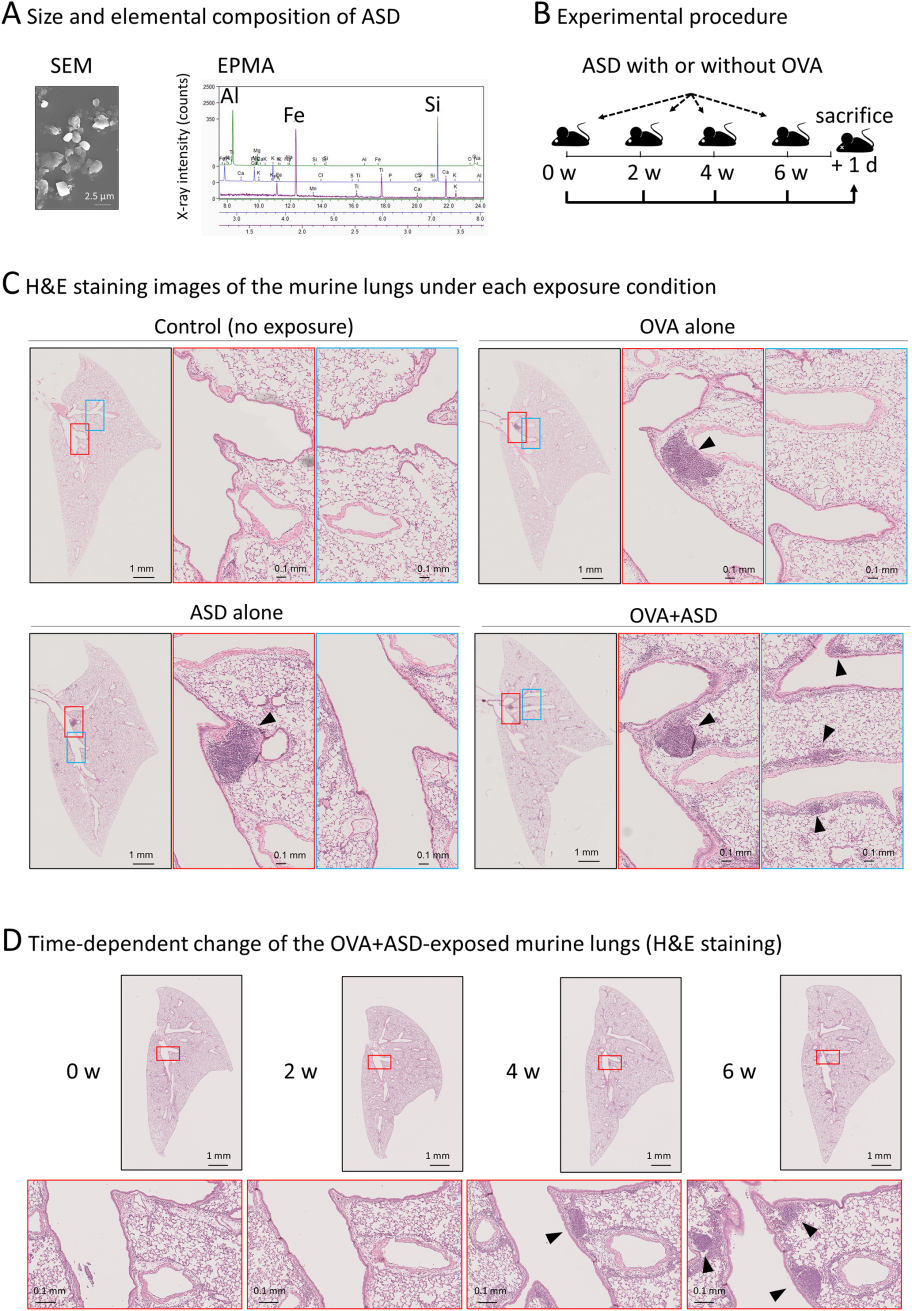
Effect of ASD and OVA-exposure on B cell-like cell cluster formation. **(A)** Scanning electron microscopy (SEM) and electron probe microanalysis (EPMA) were performed to determine the size and primary components of the ASD particles used in this study. The particles had an average diameter of approximately 2.5 µm and were primarily composed of aluminum (Al), iron (Fe), and silicon (Si), consistent with the typical composition of ASD analysis of ASD using in this study. **(B)** Experimental procedure. Six-week-old mice were intratracheally injected with ASD and/or OVA. Injections were performed four times at 2-week intervals. The day after the fourth injection, mice were sacrificed. Other details of the experiment are included in Materials and Methods. ASD, Asian Sand Dust; OVA, Ovalbumin. **(C)** Hematoxylin and eosin (H&E) staining images of the lung of each treated mouse. The lungs of the mice treated as shown in **(B)** were stained and observed under a light microscope. The section containing the largest B cell like cell cluster is shown. In samples where notable B cell like cell cluster was not observed (control), the central section is presented. ASD induced B cell-like cell cluster formation (arrowheads), which was enhanced by OVA. **(D)** Observation of OVA+ASD-induced B cell-like cell cluster formation over time. The lungs of OVA+ASD-treated mice were observed over time. The mice were treated as shown in **(B)**, and H&E staining was performed at each time point. B cell-like cell clusters appeared after 4 weeks and then increased in number and size (arrowheads).

Animal experiments were approved by the Institutional Animal Ethics Committee of Niigata University or Kyoto University and all experiments were performed in accordance with the relevant guidelines and regulations (approval number: SA00808- 2019, 2022-17, 2023-17). BALB/c and C57BL/6J mice were purchased from CLEA Japan Inc. (Osaka, Japan). Information about the CD4-Cre Bcl6^f/f^ mice is described in a previous report (14, 18). ASD was purchased as a reference material (CRM No. 30, Gobi Kosa dust) from the National Institute for Environmental Studies (Ibaraki, Japan). Although it is possible that batch-to-batch differences in ASD may affect lung pathology, likely due to variations in attached substances or elemental composition, we used a certified reference material (CRM No. 30, Gobi Kosa dust, National Institute for Environmental Studies, Japan), which is standardized and quality-controlled. Therefore, variability in composition and pathological effects was minimized in this study. OVA was used as an allergen to generate a model of the allergic diseases. Conventional murine models of allergic airway inflammation typically involve intraperitoneal sensitization with OVA prior to intratracheal OVA challenge. This sensitization step is thought to enhance immune responsiveness and prevent the development of immune tolerance, which may otherwise occur in the absence of systemic priming. However, intraperitoneal administration does not recapitulate physiologic routes of allergen exposure in natural settings. To better mimic environmentally relevant sensitization, we employed a protocol that omits intraperitoneal priming and relies solely on repeated airway exposure to OVA. This approach did not result in immune suppression or tolerance, and has been previously validated as a representative model of allergic airway disease, including asthma (19, 20). Nine-week-old male BALB/c, ten-week-old male C57BL/6J, or ten-week-old male CD4-Cre Bcl6^f/f^ mice were anesthetized with 4% isoflurane and intratracheally injected with 80 μL 0.9% NaCl solution containing 50 μg ASD with or without 4 μg ovalbumin (OVA; Grade VII, Sigma-Aldrich Co., St. Louis, MO, USA) using a 1 mL syringe fitted with a thin plastic needle. The trans-airways of each mouse were exposed to ASD with or without OVA four times at two-week intervals (**Figure 1A**). The dose of 50 µg ASD was selected based on previous *in vivo* studies showing that this amount induces measurable lung inflammation without severe toxicity in mice. It has also been used in allergic airway models involving co-exposure with OVA (3, 4). In our preliminary experiments, 50 µg consistently induced B cell cluster formation without causing extensive tissue damage. The day after each injection, the mice were anesthetized with 3% isoflurane, or pentobarbital and medetomidine hydrochloride, and then cardiac perfusion was performed with 0.9% NaCl solution and 4% paraformaldehyde (PFA) solution in 0.1 M phosphate buffer (PB; pH 7.4). In addition, the alveoli were inflated by intratracheal injection of 4% PFA solution in 0.1 M PB, and the trachea was ligated by surgical suture. After isolation from sacrificed mice, the lungs connected to the ligated trachea were refixed with 4% PFA solution in 0.1 M PB at 4°C for 12 h. Fixed samples were observed using light microscopy or scanning electron microscopy (SEM). For each exposure condition, five mice were used in each experiment. Consistent results were obtained from the same experimental groups of mice throughout the study. Representative samples are shown in the figures and tables. Additionally, nSS3D analysis was performed on one of the representative mice.

### Preparation of paraffin-embedded samples

For paraffin embedding, fixed samples were washed with 0.9% NaCl solution at 25°C for 20 min and dehydrated using a series of graded ethanol solutions (70%, 70%, 80%, 90%, 95%, 100%, 100%, and 100%) at 25°C for 3 h each. Dehydrated samples were soaked in a mixture of ethanol, chloroform, and 100% chloroform (twice) at 25°C for 30 min each. The samples were then soaked in a mixture of chloroform and paraffin at 60–70°C for 30 min and then in paraffin at 60–70 °C for 3 h (thrice). Finally, the samples were cooled to embed them in paraffin.

### Sectioning

Serial sections (6 µm thickness) were cut from paraffin-embedded samples using a sliding microtome (ROM-380, Yamato Koki Co. Ltd., Saitama, Japan) and mounted onto glass slides (CREST, Matsunami Glass, Osaka, Japan), as shown in **Supplementary Figure 1**. Three hundred serial paraffin sections were mounted on glass slides by repeating the sectioning procedure. Category 1 of the sections shown in **Supplementary Figure 1** was used for hematoxylin and eosin (H&E) staining to observe the lungs. Categories 2–5 of the sections were used for immunohistochemical staining.

### H&E staining

H&E staining was performed as previously reported (21). Briefly, paraffin-embedded sections were deparaffinized with xylene (twice) and rehydrated using a series of graded ethanol solutions (100%, 95%, 90%, 80%, and 70%) at 25°C for 10 min each. After washing with running tap water for 5 min, the sections were stained with Mayer’s hematoxylin (Fujifilm Wako, Osaka, Japan) at 25°C for 15 min. Next, the sections were washed with running tap water at 25°C for 15 min and stained with eosin (Muto pure chemicals co., ltd., Tokyo, Japan) for 3 min. Then, the sections were dehydrated using 95% ethanol (twice), 100% ethanol for a few seconds (twice), and xylene at 25°C for 1 h (thrice). The dehydrated sections were mounted with a mounting medium and dried at 25°C

### Immunohistochemical staining

Paraffin-embedded sections were deparaffinized with xylene and rehydrated using a series of graded ethanol solutions. The sections were soaked in 10 mM sodium citrate buffer (pH 6.0), and antigenicity was retrieved by preheating at 121°C for 20 min in an autoclave. After washing the sections with phosphate-buffered saline (PBS) at 25°C for 3 min, endogenous peroxidase activation was blocked by incubation with 0.3% hydrogen peroxide solution at 25°C for 10 min. After washing the sections with PBS at 25°C for 3 min, non-specific binding of antibodies was blocked by incubation for 30 min at 25°C in a solution containing 4% Block Ace (Yukijirushi, Hokkaido, Japan). After washing in PBS at 25°C for 3 min, the sections were incubated with primary antibodies (F4/80: #30325, CD11c: #97585, CD19: #90176, CD3: #85061, Ki67: #62548; Cell Signaling Technology, Danvers, MA, USA; B220: 14-0452-82; Thermo Fisher Scientific, MA, USA) diluted in 1% bovine serum albumin in a humidified chamber at 4°C for 12 h. After washing the sections with PBS-T (PBS with 0.05% Tween 20) at 25°C for 5 min, non-specific binding of antibodies was blocked by incubation for 30 min at 4°C in a humidified chamber using 10% normal goat serum from the species of the secondary antibody (Histofine SAB-PO (R) kit, Nichirei, Tokyo, Japan). The sections were then incubated with a goat anti-rabbit or anti-rat IgG-HRP secondary antibody (Histostar, MBL, Nagoya, Japan) in a humidified chamber at 4°C for 30 min. After washing the sections twice with PBS-T at 25°C for 5 min, HRP activity was visualized by incubation in 0.05% 3,3′-diaminobenzidine solution (Histofine SAB-DAB kit, Nichirei). The sections were then counterstained with Mayer’s hematoxylin at 25°C for 15 min, washed with running tap water for 15 min, dehydrated with 95% ethanol (twice), 100% ethanol for a few seconds (twice), and xylene for 1 h (thrice). The dehydrated sections were mounted with a mounting medium and dried at 25°C.

### Next generation serial section-three-dimensional reconstruction

Images were acquired using an automatic virtual slide system (NanoZoomer S210, Hamamatsu Photonics K.K., Shizuoka, Japan). The serial section images of bronchi and vessels and those of B cells were obtained from H&E and immunohistochemical staining images, respectively. The positions and boundary inclinations of the serial section images were adjusted using software we developed. In this study, image alignment was performed using two registration steps: “rigid registration” and “non-rigid registration”. Rigid registration corrects global misalignment between serial sections by adjusting for translation and rotation, assuming no deformation of tissue shape. In contrast, non-rigid registration allows for local deformations to improve alignment accuracy. These procedures were implemented using in-house AI-based software. Due to confidentiality issues, the software is not publicly available. In brief, rigid and then no-rigid registration was performed using models based on Valis (22) and AiR (23), respectively. No-rigid registration involved cropping each image to tiled images and dividing them in the ratio “train:validation:test = 3:1:1.” These tiled images were used for learning and estimating the model. To support accurate alignment of serial sections, we used a two-layer convolutional neural network (CNN) to identify and match similar regions across adjacent images. This model was applied during a step called “patch embedding,” in which small image tiles are converted into numerical representations based on their visual features. To improve the quality of alignment, we also added a regularization term to the model’s loss function. This term was based on normalized cross-correlation (NCC) (24), a method commonly used to assess the similarity between image patches. Incorporating NCC helped the model align structures with greater precision. For 3D reconstruction of bronchi, vessels, and B cells from 2D images, the colored digital images were loaded into proprietary modified software based on Amira (Thermo Fisher Scientific, MA, USA). Briefly, the following process was applied: (і) Align Slices, (ii) Extract Subvolume, (iii) Interactive Thresholding, (iv) Binary Smoothing, (v) Generate Surface, and (vi) Surface View.

### Scanning electron microscopy

Samples were prepared as previously described (25). Briefly, samples fixed with 4% PFA were additionally fixed with a 2% glutaraldehyde solution in 0.1 M PB (pH 7.4) at 4°C for 12 h. After washing thrice in 0.1 M PB at 25°C for 20 min, the samples were subjected to conductive staining with 1% tannic acid (Nacalai Tesque, Kyoto, Japan) at 25°C for 2 h. After washing thrice in 0.1 M PB at 25°C for 20 min, the samples were post-fixed with 1% osmium tetroxide solution in 0.1 M PB (pH 7.4) at 25°C for 2 h. After conductive staining, they were dehydrated in a series of graded ethanol solutions (70%, 70%, 80%, 90%, 95%, 100%, 100%, and 100%) at 25°C for 3 h each, substituted with isoamyl acetate, and dried in a critical point dryer (HCP-2, Hitachi, Tokyo, Japan). The dried samples were mounted on a metal plate with conductive tape and coated with platinum–palladium using an ion-sputter coater (E1010, Hitachi). The samples were observed by SEM (S-4300, Hitachi) at an accelerating voltage of 10 kV.

## Results

### Effect of exposure to ASD with or without OVA on B cell-like cell cluster formation in murine lung

Initially, we confirmed the size and elemental composition of the ASD used in this study. Scanning electron microscopy (SEM) and electron probe microanalysis (EPMA) revealed that the ASD particles have an average diameter of approximately 2.5 µm, and are primarily composed of aluminum (Al), iron (Fe), and silicon (Si), consistent with the composition of general ASD reported previously (5) (**Figure 1A**). Experimental procedure of ASD-exposure with or without OVA was shown in **Figure 1B**. To investigate the effect of ASD on B cell cluster formation in the presence of pulmonary allergic diseases, OVA was used as an allergen to generate a model of the allergic diseases. Each left lung sample was thinly sliced, and then stained with H&E. The section containing the largest B cell-like cell cluster is shown in **Figure 1C**. B cell-like cell clusters were scarcely observed in control (no exposure) mice, but were observed in OVA alone, ASD alone, and OVA+ASD-exposed mice (**Figure 1C**). Although there was no marked difference between OVA alone, ASD alone, and OVA+ASD-exposed mice in the level of development of the largest B-cell like cell cluster formed in the main bronchial branch site (**Figure 1B**, middle panels, arrowheads), small B-cell like cell clusters appeared to be formed around branched blood vessels and bronchi in the OVA+ASD-exposed mice (**Figure 1C**, right panels, arrowheads). Since OVA+ASD mice seemed to be the most strongly affected by exposure, the development of changes over time was observed for this group. B cell-like cell clusters were not observed at week 2 but were observed at week 4 and increased in number at week 6 in OVA+ASD-exposed mice (**Figure 1D**, arrowheads).

### Histological structure of the OVA+ASD-induced B-cell like cell clusters

**Figure 2:**
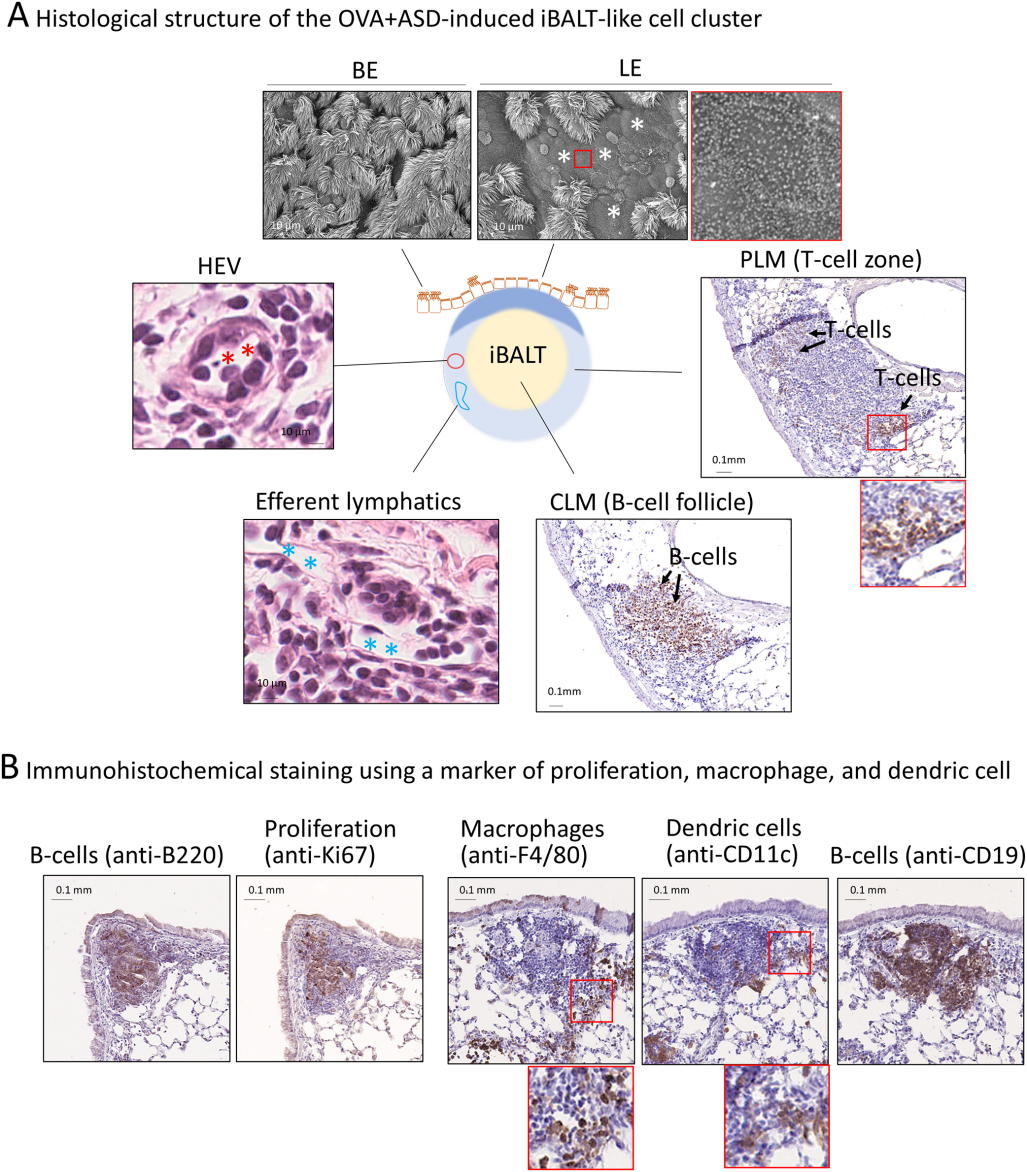
Structure of the largest OVA+ASD-induced B cell like-cell cluster around main bronchus. **(A)** BALT comprises lymphoepithelium (LE), central lymphoid mass (CLM), and peripheral lymphoid mass (PLM) (26). The LE and peripheral bronchial epithelium (BE) were observed using scanning electron microscopy (upper panels). LE comprises cuboidal epithelial cells with microvilli. The CLM and PLM were observed using immunohistochemical staining with anti-CD3 (a marker of T-cells) and anti-B220 (a marker of B cells) antibodies, or H&E staining. Immunohistochemical staining showed that the CLM and PLM primarily consist of B and T-cells, respectively (right and right-lower panels). H&E staining showed that the cell cluster contains high endothelial venules (HEVs) (red asterisks) and well-developed efferent lymph vessels (blue asterisks) (left and left-lower panels). **(B)** Observation of proliferating cells, macrophages, and dendritic cells. The cell cluster was imaged after immunohistochemical staining using anti-Ki67 (a marker of proliferation), anti-F4/80 (a marker of macrophages), anti-CD11c (a marker of dendritic cells), anti-B220, and anti-CD19 (B cell markers) antibodies. B cells, which make up the majority of the CLM, are proliferative cells (left two panels). The PLM contains macrophages and dendritic cells (right three panels) in addition to T-cells (**A**, right panel).

To confirm whether the OVA+ASD-induced B-cell like cell clusters were iBALT, we performed histological characterization. iBALT has been reported to be an aggregation of different types of cells, mainly B cells; it is associated with the bronchi and contains the following regions: lymphoepithelium (LE) at the mucosal surface, central lymphoid mass (CLM) at the center of the aggregation, and peripheral lymphoid mass (PLM) surrounding the CLM (**Figure 2A**) (26). While traditional BALT is typically associated with the overlying epithelium characterized by microvilli, iBALT often forms independently of the airway epithelium and can be located in distal regions of the lung (13). Therefore, in this study, we first analyzed the largest B cell-like cell cluster induced by combined OVA and ASD exposure, because it was consistently observed adjacent to the bronchial epithelium at the site of the main bronchial bifurcation. SEM revealed that, whereas the bronchial epithelium (BE) was primarily composed of ciliated cells, LE of the largest B cell-like cell clusters consisted of epithelial cells bearing microvilli (**Figure 2A**, upper panels). H&E staining and immunohistochemical staining using antibodies against each cell marker revealed that the CLM in the largest B-cell like cell cluster was an aggregation of lymphocytes, mainly B cells (**Figure 2A**, right-lower panel), and the PLM mainly comprised T cells (**Figure 2A**, right panel), but also contained high endothelial venules (HEVs) (**Figure 2A**, left panel) and well-developed efferent lymphatic vessels (**Figure 2A**, left-lower panel). Furthermore, immunohistochemical staining showed the proliferation of B cells in the CLM and dendritic cells (DCs) and macrophages (26) in the PLM (**Figure 2B**).

### Next generation serial section 3D imaging of OVA+ASD-induced iBALT

**Figure 3:**
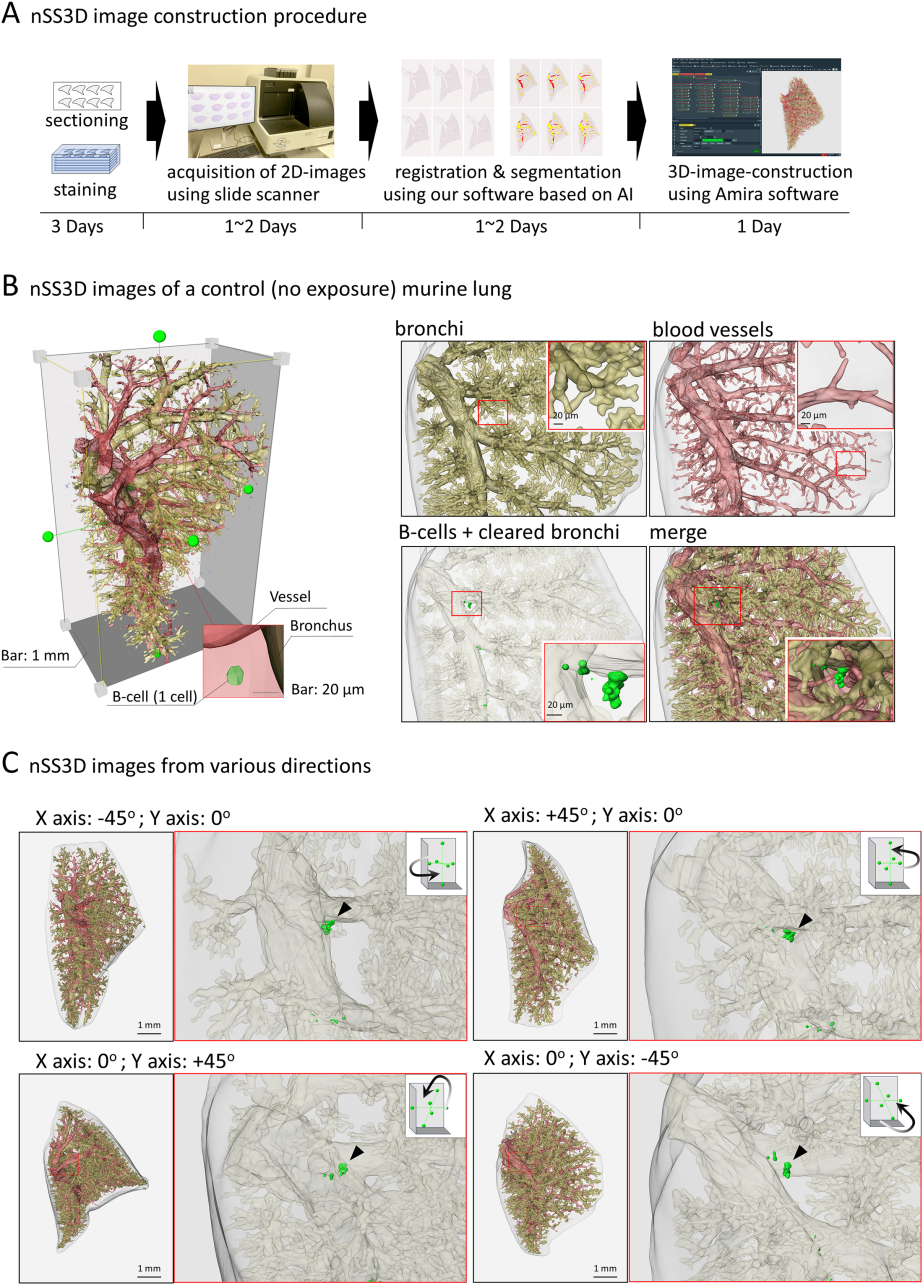
Next-generation serial section-three-dimensional (nSS3D) images of murine lung. **(A)** Schematic images of nSS3D image construction procedure, using 300 serial sections (6 µm thickness). After H&E and immunohistochemical staining using anti-B220 antibodies, 2D images of the serial sections were obtained via a high-resolution slide scanner (max 0.23 μm/pixel). The 2D images were then registered and segmented using our software. The 3D images were constructed using our modified Amira software. Other details of the experiment are included in Materials and Methods. **(B)** nSS3D images of a control (no exposure) murine lung. An overall view of the murine left lung was constructed using nSS3D imaging technology (left panel). The right panel shows the respective and merged images of the bronchus, blood vessels, and B cell clusters. nSS3D images allowed detailed observation of vascular and bronchial conditions. In addition, B cell clusters could be observed in the lungs of non-treated mice, albeit on a small scale, unlike in 2D images after H&E or immunohistochemical staining. **(C)** nSS3D images from various directions. The left panels show merged images of the vessels, bronchi, and B cells of the whole left lung of a mouse, acquired as indicated in **(A)**. The right panels show enlarged images of the bronchi and B cells. A small B cell clusters could be observed at the site of a bronchial branch from the main bronchus.

**Figure 4:**
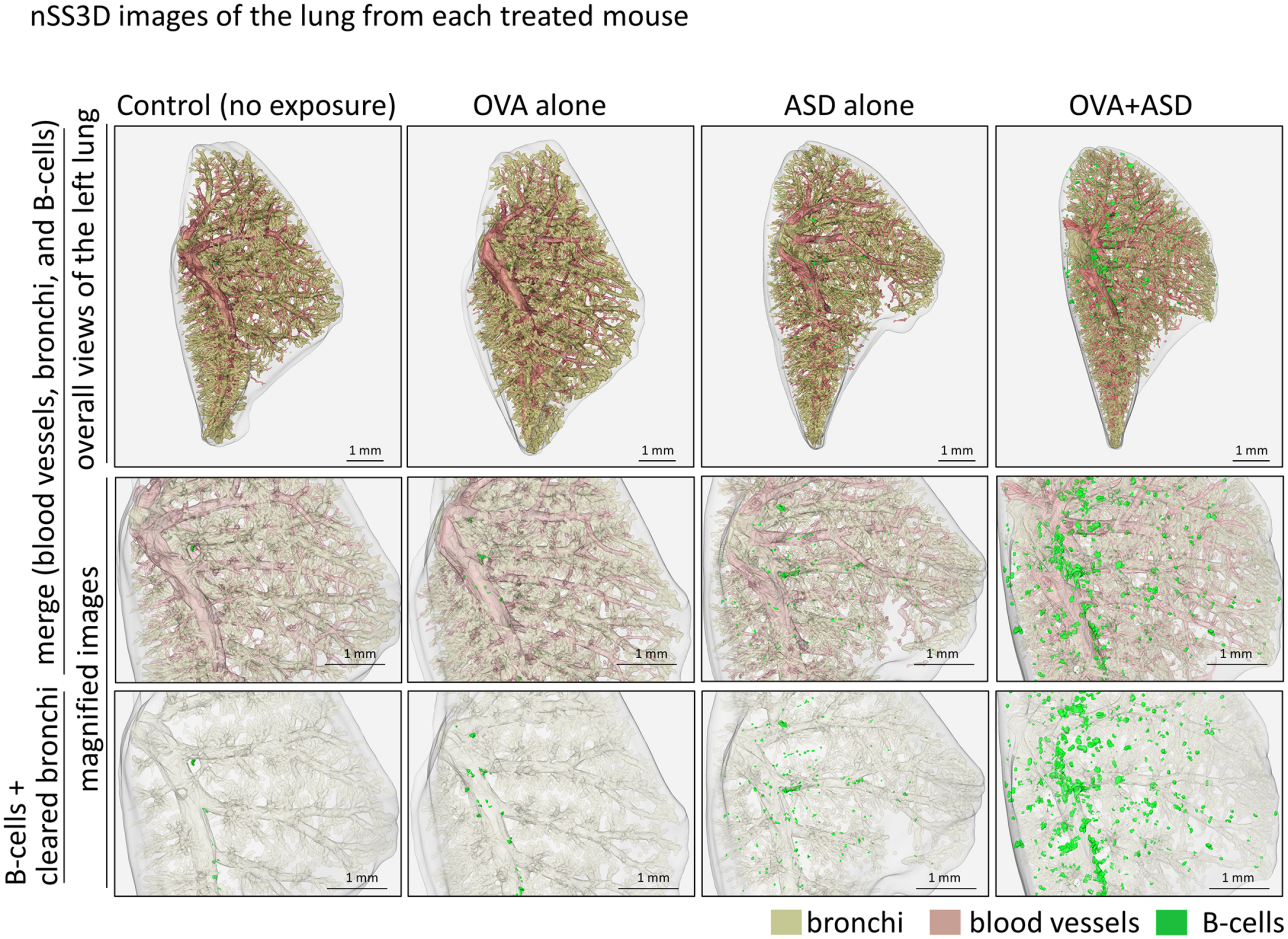
nSS3D images of the lung from each treated mouse. B cell cluster formation can be observed more clearly in the nSS3D image than in the 2D image in **Figure 1**. The number of B cell clusters was higher in the ASD-treated mouse than in the untreated control, and this was enhanced by OVA.

We next attempted to analyze the OVA+ASD-induced B cell clusters in more detail, in which the applicability of nSS3D technology for the analysis of mouse lungs was assessed. First, we tested whether the nSS3D method could be used to construct 3D images of blood vessels, bronchi, and B cells in the mouse lung within the same field of view by combining H&E-stained images with immunohistochemically stained images. Approximately 300 sections/specimen were used for nSS3D image construction. The section images were registered and segmented using our AI-based software (**Figure 3A**). An overall view of the left lung of a control mouse is shown in **Figure 3B** (left panel), along with magnified images of each component (blood vessels, bronchioles, and B cells (**Figure 3B**, right panels). **Figure 3C** presents a view of the B cell signal and the surrounding tissue from multiple angles. These nSS3D images demonstrate that it is possible to reconstruct bronchi in three dimensions at the level of fine or terminal bronchioles, and for blood vessels, at the level of 20~50 µm in diameter (**Figure 3B**, right panels). In addition, the 2D H&E-stained images scarcely showed B cell clusters in the lungs of the control mice (**Figure 1**), whereas the nSS3D images revealed their presence, albeit to a slight extent (**Figures 2B, C**). Moreover, images from multiple angles show that the small B cell cluster is located on the acute side of the bronchial branching site from the main bronchus (**Figure 3C**). The diameter corresponding to the smallest signal for B cells is approximately 20 µm, which can be detected at the single-cell level. nSS3D imaging was next used to analyze the left lung of each type of mouse exposed as shown in **Figure 1** (**Figure 4**). More B cell clusters were observed in the OVA alone, ASD alone, and especially the OVA+ASD-exposed mice compared to the control mice, with the OVA+ASD-exposed mice exhibiting the most pronounced development. Furthermore, in the OVA alone and ASD alone-exposed mice, B cell clusters were primarily observed around the large bronchi. In contrast, in the OVA+ASD-exposed mice, these clusters were also frequently found around blood vessels and within the interstitial tissue distant from the bronchi. These ectopic clusters appeared to be smaller in size than those located around the main bronchus.

**Figure 5:**
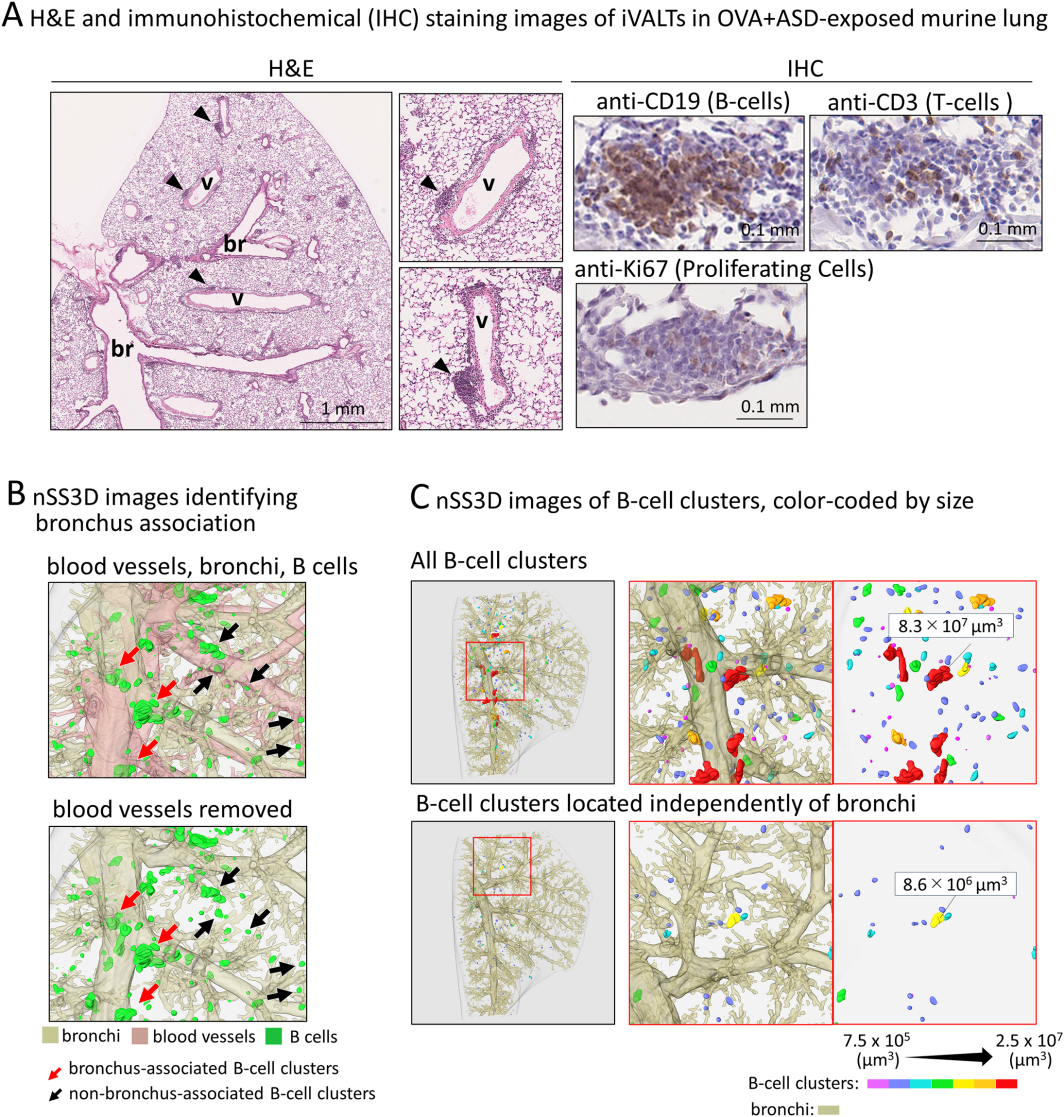
Analysis of size and localization of the OVA+ASD-induced B cell clusters. In this study, for convenience, B cell clusters formed around the blood vessels and zonal bronchi region are referred to as iVALTs. **(A)** Images after H&E (left) and immunohistochemical staining using antibodies against the indicated markers (right). br: bronchus; v: blood vessel. iVALTs are seen in association with blood vessels (arrow heads). iVALTs mainly comprise B and T-cells, but there is no compartmentalization of the two. In addition, few cells show Ki67 positivity, a marker for proliferative cells, and their signal is weak in iVALTs. **(B)** Identification of B cell clusters including iVALTs by nSS3D imaging. Upper panel: bronchus, blood vessel, and B cells. Blood vessels were deleted in the lower image, demonstrating that iVALT is associated with blood vessels and not bronchi. **(C)** Identification of B cell clusters by size and distance from the nearest bronchus. The upper panel shows all B cell clusters color-coded by size. The bottom panel shows a collection of B cell clusters more than 200 µm away from the nearest bronchus. nSS3D analysis confirms that iVALT is not associated with bronchi.

**Analysis of the localization and size of B cell clusters induced by OVA+ASD- exposure** Most B cell clusters were observed around the main bronchus in the lungs of mice exposed to OVA alone and ASD alone, but signals of B cell markers forming small cluster were also scattered throughout the lung and observed around the blood vessels and zonal bronchi region in OVA+ASD-exposed mice. Therefore, a more detailed analysis of these B-cell clusters was performed. Herein, for convenience, B cell clusters formed around the blood vessels and zonal bronchi region are referred to as inducible vessel-associated lymphoid tissues: iVALTs, analogous to iBALTs. 2D images of H&E-stained sections showed that iVALTs were more prevalent in the perivascular area than in the bronchi (**Figure 5A**, left panels, H&E). Furthermore, they appeared to be smaller than the iBALT-like clusters around the main bronchus shown in **Figures 1** and **2**. Immunohistochemical staining was then performed. The primary cells comprising iVALTs were B cells, as in iBALTs, with T-cells sparsely scattered throughout (**Figure 5A**, right panel, IHC). There was no Ki67 signal indicating proliferating cells; if present, it was weak. 2D images of H&E-stained sections showed that iVALTs were associated with blood vessels; however, it is not clear whether they are also associated with bronchi. We then investigated the ability of the nSS3D method to discriminate between B cell clusters associated with and not associated with bronchi. The nSS3D images clearly showed the presence of perivascular iVALTs independent of the bronchi (**Figure 5B** left panels). Next, to quantify B-cell clusters associated with and not associated with bronchi separately, we measured the number and size of B cell clusters that were more than 200 µm away from the nearest bronchus and those that were not, in which small clusters were excluded from the analysis (0.75 × 10^6^ µm^3^, i.e., approximately 1,000 cells or less in size) (**Figure 5B** right panels). Using the distance from the bronchus as a limiting condition, B cell clusters around bronchi and those located independently of them could be distinguished from each other. Both types of B-cell clusters in the lungs of OVA+ASD-exposed mice are color-coded by size and shown in **Figure 5B**. The large B-cell clusters, indicated in red, are not observed in the cell clusters away from bronchi. Additionally, the same analysis was performed for the other experimental groups, and the quantitative results based on their nSS3D images are presented in **Table 1**. OVA+ASD-exposed mice had the highest numbers of B-cell clusters around and away from bronchi, with 363 and 86, respectively. ASD alone-exposed mice ranked second, with 10 B cell clusters around bronchi and 1 cluster located independently of them, followed by OVA alone, with 5 B cell clusters around bronchi and no bronchus-independent clusters. A similar trend was also observed for the total volume of B-cell signals and mean volume of B cell clusters around bronchi. For B-cell clusters located independently of bronchi, the mean volume was 1.8 µm³ for OVA+ASD, which was smaller than that of the B cell clusters around bronchi (**Table 1**).

**Table 1:** Quantitative analysis of B-cell clusters in mice under each exposure condition based on nSS3D images.

Exposure condition	None	OVA alone	ASD alone	OVA+ASD
Total volume of B-cell signals (fold of none-exposed control)	1	3.2	11.8	136.6
B cell clusters around bronchi (number)	1	5	10	363
B cell clusters away from bronchi (number)	0	0	1	86
Mean volume of B cell clusters around bronchi iBALT (µm^3^)	–	1.2	1.9	3.4
Mean volume of B cell clusters away from bronchi (µm^3^)	–	–	–	1.8

### Effect of follicular helper T cell deficiency on OVA+ASD-induced B-cell cluster formation

**Figure 6:**
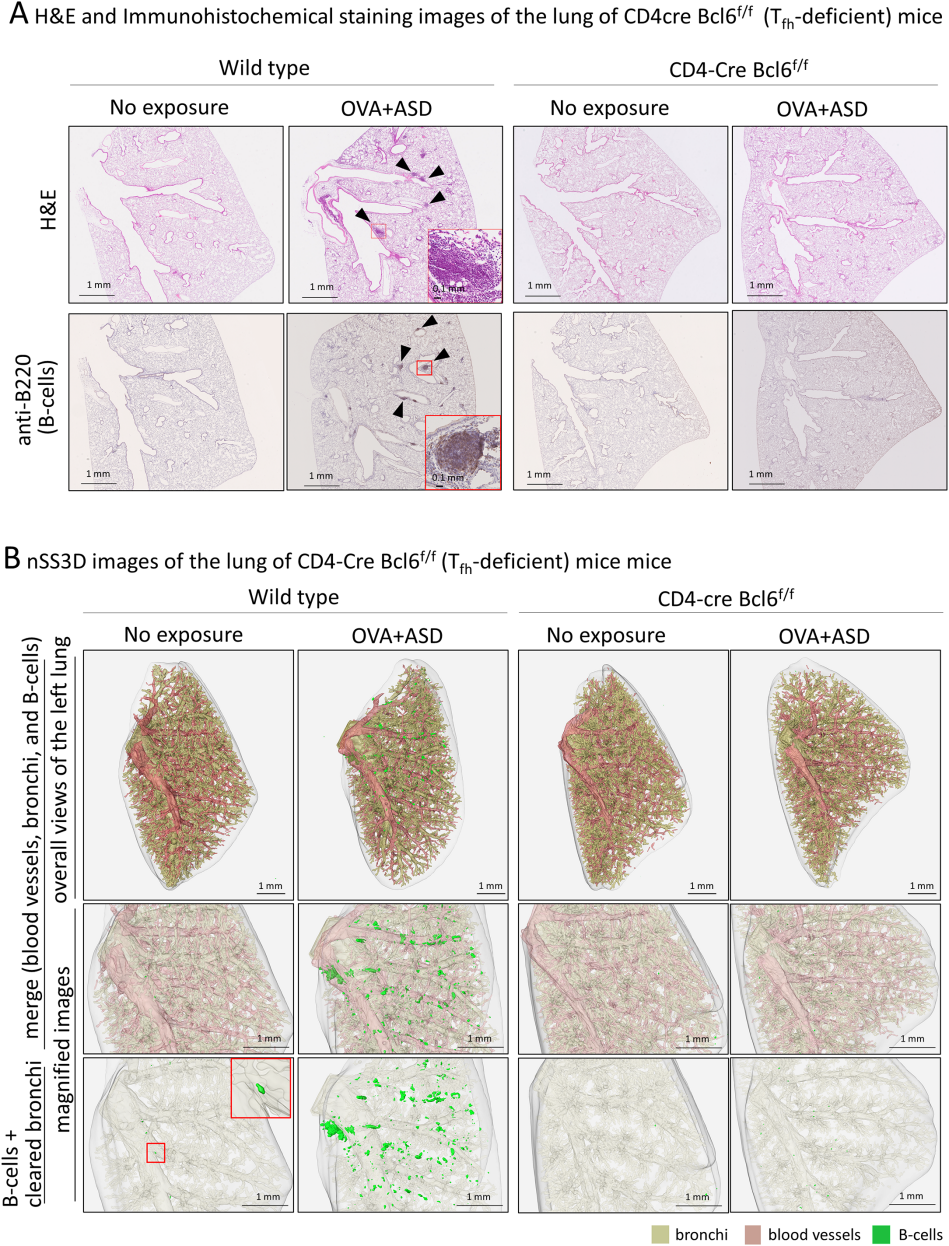
Effect of T follicular helper (T_fh_) cell deficiency on OVA+ASD-induced B cell cluster formation. Since infection with *Mycobacterium tuberculosis* has been reported to induce iBALT formation via T_fh_ cells, effects of T_fh_ cell deficiency on OVA+ASD-induced B cell cluster formation were investigated using CD4-Cre Bcl6^f/f^ mice lacking T_fh_ cells. CD-4Cre Bcl6^f/f^ and wild-type mice were treated with or without OVA+ASD, and then their lungs were observed. **(A)** Images after H&E (upper panels) and immunohistochemical staining using anti-B220 (lower panels). OVA+ASD induced B cell clusters (arrowheads) in the lungs of wild-type mice. In contrast, OVA+ASD failed to induce the formation of B cell clusters in the lungs of CD4-Cre Bcl6^f/f^ mice. **(B)** The 3D image of the lung shown in **(A)** was constructed using the nSS3D method. The upper panels show an overall view of the left lung with merged blood vessels, bronchi, and B cell clusters; the middle panels show magnified images of the central portion; and the lower panels show B cell clusters with cleared bronchi in the middle portion. Similar to **(A)** but more clearly, nSS3D images show that OVA+ASD induced B cell cluster formation (green) in the lungs of wild-type mice but not CD4-Cre Bcl6^f/f^ mice.

T_fh_ cells are a subset of CD4-positive helper T cells involved in humoral responses (6). They are found in secondary lymphoid tissues such as the tonsils, spleen, and lymph nodes. Their role in these tissues is to activate B-cells (7, 8). Therefore, T_fh_ cells may be involved in the formation of B cell clusters induced by OVA+ASD, at least in those exhibiting iBALT-like structures, as shown in **Figure 2**. To test this possibility, we analyzed CD4-Cre Bcl6^f/f^ mice lacking T_fh_ cells (14). Since the CD4-Cre Bcl6^f/f^ mice are C57BL/6J line, the same exposure experiments were performed using wild type C57BL/6 mice as controls. 2D images of H&E and immunohistochemically stained sections with antibodies against B cell marker showed that B-cell clusters including iVALTs were scarce in the lungs of no exposure wild-type mice; however, remarkable their formation was observed after exposure to OVA+ASD (**Figure 6A**). In contrast, no OVA+ASD-induced formation of B-cell clusters was observed in CD4-Cre Bcl6^f/f^ mice (**Figure 6A**). Similar to the analysis presented in **Figure 5** and **Table 1**, the tissues were further analyzed using the nSS3D method. While conventional H&E staining did not detect any B-cell clusters in wild-type mice without exposure, nSS3D imaging and subsequent quantitative analysis revealed the presence of a few small B-cell clusters located both around and distant from the bronchi (**Figure 6B**, **Table 2**). A marked increase in both types of B-cell clusters was observed in wild-type mice following exposure to OVA+ASD (**Figure 6B**, **Table 2**). In contrast, no such increase was observed in CD4-Cre Bcl6^f/f^ mice following OVA+ASD exposure compared to non-exposed CD4-Cre Bcl6^f/f^ controls (**Figure 6B**, **Table 2**).

**Table 2:** Quantitative analysis of B-cell clusters in CD4-Cre Bcl6f/f mice with and without exposure to OVA+ASD, based on nSS3D images.

Exposure condition	WT		CD4-Cre Bcl6^f/f^	
	None	OVA+ASD	None	OVA+ASD
Total volume of B-cell signals (fold of each none-exposed control)	1	134.5	1	1
B cell clusters around bronchi (number)	3	253	4	3
B cell clusters away from bronchi (number)	1	50	1	0
Mean volume of B cell clusters around bronchi iBALT (µm^3^)	0.9	2.5	0.5	0.5
Mean volume of B cell clusters away from bronchi (µm^3^)	–	1.3	–	–

## Discussion

This study has demonstrated that ASD induces iBALT formation in mouse lung tissue. Furthermore, such induction is enhanced by OVA exposure. In the time-course observation following OVA+ASD exposure, progressive development of B cell cluster formation was observed. This suggests that in the experimental model used in this study, repeated exposure did not induce immune suppression or tolerance, but rather mimics chronic allergic diseases such as asthma. Therefore, patients with chronic conditions such as bronchial asthma may experience more severe symptoms when exposed to ASD. The lungs are exposed to numerous microorganisms and foreign matter via the bronchi. The mucosal immune system secretes immunoglobulin A (IgA) from the pulmonary mucosal surface to resist pathogens (27, 28) through the common mucosal immune system (CMIS), which comprises inductive and effector sites (29). BALT is an essential component of the CMIS that combats antigens and induces antigen-specific IgA production (29). Histological characterization of the large B cell clusters formed around the main bronchus upon exposure to OVA+ASD revealed features consistent with those previously reported for BALT (26). For example, their CLMs consist primarily of B cells, whereas their PLMs contain T cells, DCs, and macrophages. Accordingly, these findings suggest that the B-cell clusters induced by OVA + ASD, at least in part, constitute bona fide iBALT structures and may function as immune response-inducing sites. In addition, the PLMs in OVA+ASD-induced iBALTs contain HEVs (30). Trafficking of lymphocytes from the bloodstream to secondary lymphoid organs (SLOs) is essential for generating immune responses and providing an efficient defense against pathogens (31); this process is mediated by interactions between lymphocytes and HEVs in SLOs (31, 32). Thus, OVA+ASD-induced iBALT functions not only as an immunity-inducing tissue but also as a trafficking site for lung lymphocytes.

Both traditional BALT and iBALT mainly comprise B cells (26). However, in the murine lungs examined in this study, not all pulmonary B cells formed iBALTs, e.g., those in OVA+ASD mice. B cells often form clusters in the perivascular space, e.g., iVALTs; they weakly express proliferation markers and are not separated from the T cell zones. The nSS3D images obtained in this study reveal that most iVALTs are associated with blood vessels but not with bronchi. Moreover, iVALTs often cover smaller areas than iBALTs. As its name suggests, BALT is typically formed in the submucosal regions surrounding the bronchi. However, iBALT can also develop in perivascular or interstitial areas (13). Moreover, although these ectopic lymphoid structures can be functional, they do not always exhibit the classical features of traditional BALT (13). Therefore, the possibility that the iVALTs observed in this study may eventually develop into functional iBALT cannot be disregarded. However, none of the iVALTs analyzed in this study exhibited large cell clusters comparable in size to those formed adjacent to the main bronchus. If iVALTs were indeed precursors of functional iBALT, one might expect the presence of similarly large aggregates resembling peribronchial iBALT among them. This suggests that the mere presence of a small B cell cluster does not necessarily indicate the initiation of iBALT, but rather that additional microenvironmental factors or cellular interactions may be required to trigger its organized development. Our nSS3D technology has demonstrated a robust capability to detect and visualize the distribution, number, and size of B-cell clusters, including iBALTs and iVALTs, in mouse lung tissue. This enables a more precise and comprehensive characterization of these distinct lymphoid structures, which may contribute to improved understanding of their individual roles in pulmonary immune responses. Compared with traditional confocal microscopy-based 3D reconstruction, which requires fluorescent labeling and optical sectioning, nSS3D offers several advantages. It enables full-organ reconstruction using conventional histological staining (e.g., H&E or immunohistochemistry), and is less affected by optical interference from particles or pigments such as those present in environmental exposures. Additionally, nSS3D provides high-resolution, full-thickness information without the need for tissue clearing, which can be difficult when foreign material is present.

Interleukin 17 (IL-17)-producing T cells, such as Th17 cells, contribute to iBALT formation (26). Studies on IL-17- and IL-17R*-*deficient mice have revealed substantial attenuation of iBALT formation during *Mycobacterium tuberculosis* infection (33, 34). In addition, a chronic lung inflammation model established by repeated intranasal LPS administration in neonatal mice demonstrated that iBALT formation was mediated by IL-17-producing T_fh_ cells (35), in which IL-17 in the induction of C-X-C motif chemokine ligand 13 (CXCL13) was suggested to be important. In this study, OVA+ASD-induced B cell cluster formation was attenuated in T_fh_-deficient (CD4-Cre Bcl6^f/f^) mice; therefore, the same molecular mechanism involving IL-17 may play an important role in the B cell cluster induction by OVA+ASD. Interestingly, not only large B-cell clusters around the main bronchus, presumably functional iBALTs, but all B-cell clusters including iVALTs were absent in T_fh_-deficient mice, even under OVA+ASD exposure. Although more detailed analysis is required, this result may suggest that T_fh_ cells are involved at an earlier stage of iBALT development, i.e., in the recruitment of B cells to the lung tissue, rather than in the formation or maintenance of higher-order iBALT structures. Furthermore, in a modified vaccinia virus Ankara infection model, iBALT structures were induced in an IL-17-independent but CXCL13-dependent manner (36). In addition, CXCL13-independent but IL-1-dependent iBALT formation has been observed in the lungs of mice after influenza virus infection (37). Thus, multiple mechanisms may regulate iBALT formation depending on the type of pathogen. In this context, we have previously found that the intratracheal injection of fine particles such as aluminum salts and silica kills alveolar macrophages, which subsequently release IL-1α and induce the formation of iBALT in the murine lung. Interestingly, the ASD used in this study also contained Al and Si as its main components. Additionally, B cell clusters induced by OVA+ASD exposure are accompanied by the infiltration of F4/80-positive cells (macrophages) around blood vessels and bronchi. In CD4-Cre Bcl6^f/f^ mice, this macrophage infiltration disappears along with B cell clusters (**Supplementary Figure 2**). Therefore, the T_fh_–macrophage–B cell cluster including iBALT and iVALT axis may be a potential therapeutic target for allergic inflammation exacerbated by ASD.

Finally, this study demonstrates that our nSS3D analysis technique is effective for the pathological evaluation of murine lung tissue. Although two-dimensional observation of tissue sections yielded variable results depending on the section, and B cell clusters could not be identified in the Control group, nSS3D clearly shows the differences in B cell cluster formation between the respective experimental groups, more so than 2D images following H&E and/or immunohistochemical staining. Furthermore, the levels of B cell clusters induced by OVA+ASD were successfully identified and quantified using nSS3D analysis. However, the same conclusions about the effect of ASD on B cell cluster formation, based on nSS3D analysis, could have potentially been drawn using less intensive and time-consuming histopathological methods that surveyed larger areas of the lung in more mice with less detail, placing greater focus on the peripheral regions of the lung. Indeed, our nSS3D technology enables analysis with dramatically higher precision and in a shorter time than conventional manual human analysis. However, it still takes approximately one week to analyze a single left lung of a mouse. At present, nSS3D does not offer the high-throughput convenience like a simple pathology test. Nevertheless, itis clear that nSS3D is capable of more detailed analysis. For example, in this study, H&E-stained images did not clearly detect B cell clusters in control mice, but nSS3D did. Additionally, counting B cell clusters around bronchi and iVALT throughout the whole lung and quantifying their volumes are challenging with conventional two-dimensional analysis. Furthermore, various types of 2D images can be combined in nSS3D analysis. Hence, nSS3D analysis is expected to be applied not only to B cell clusters including iBALTs and iVALTs of lung tissue, but also to the analysis of various organs in the future. Therefore, it is important to choose between these methods depending on the purpose of the analysis and the situation. Further advancements in nSS3D technology based on the results of this study are expected to make it even more useful in the future.

In conclusion, present study strongly suggests that ASD exacerbates allergic disease and that B-cell clusters including iBALTs and iVALTs may be involved there. in addition, characterization of the OVA+ASD-induced B cell clusters proved that the nSS3D technique is useful for the analysis of mouse models. Development of B cell clusters including iBALT and the underlying mechanisms of function vary among various diseases. Hence, it is a potential target for immune system research on various lung diseases. The results of this study will help advance such research. They also emphasize the need for medical countermeasures for patients with asthma and other allergic diseases living in areas with environmental particulate matter, such as ASD, contamination.

## Data Availability

The raw data supporting the conclusions of this article will be made available by the authors, without undue reservation.
