# Supplementary figures and images for: Characterization of bronchus-associated lymphoid tissue induced by co-exposure to Asian sand dust and ovalbumin: a study using 3D serial section imaging
Source: Front Immunol. 2025 Aug 4;16:1578255. doi: 10.3389/fimmu.2025.1578255 (PMC12358379; doi:10.3389/fimmu.2025.1578255)

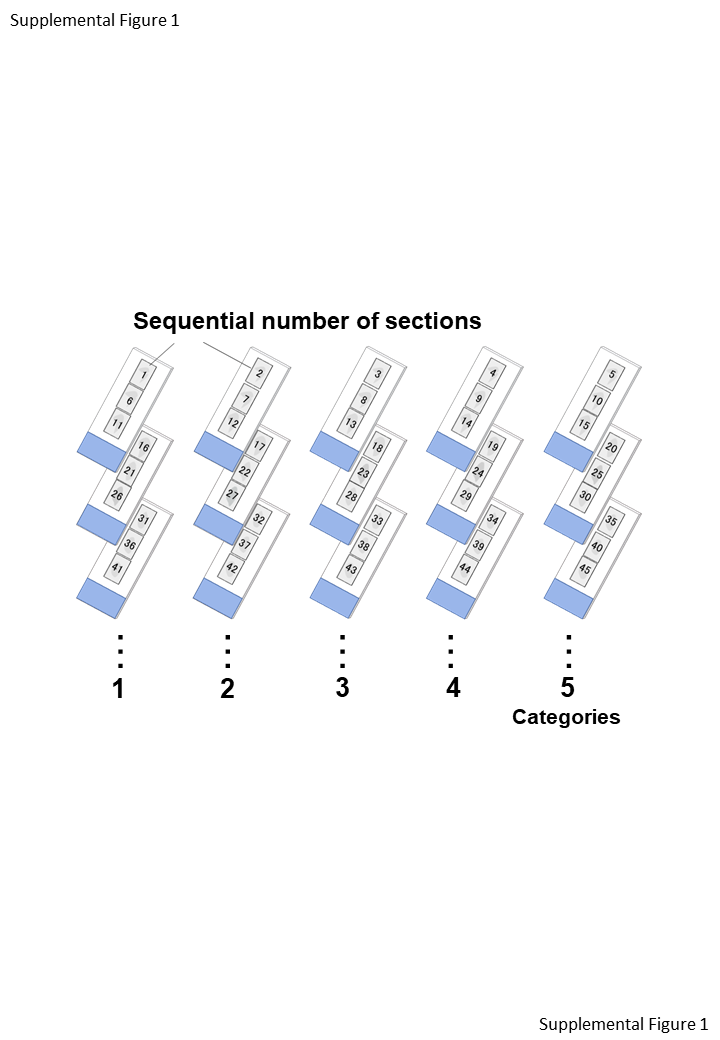

Supplement: Supplementary file 1 [file Image1.tif]

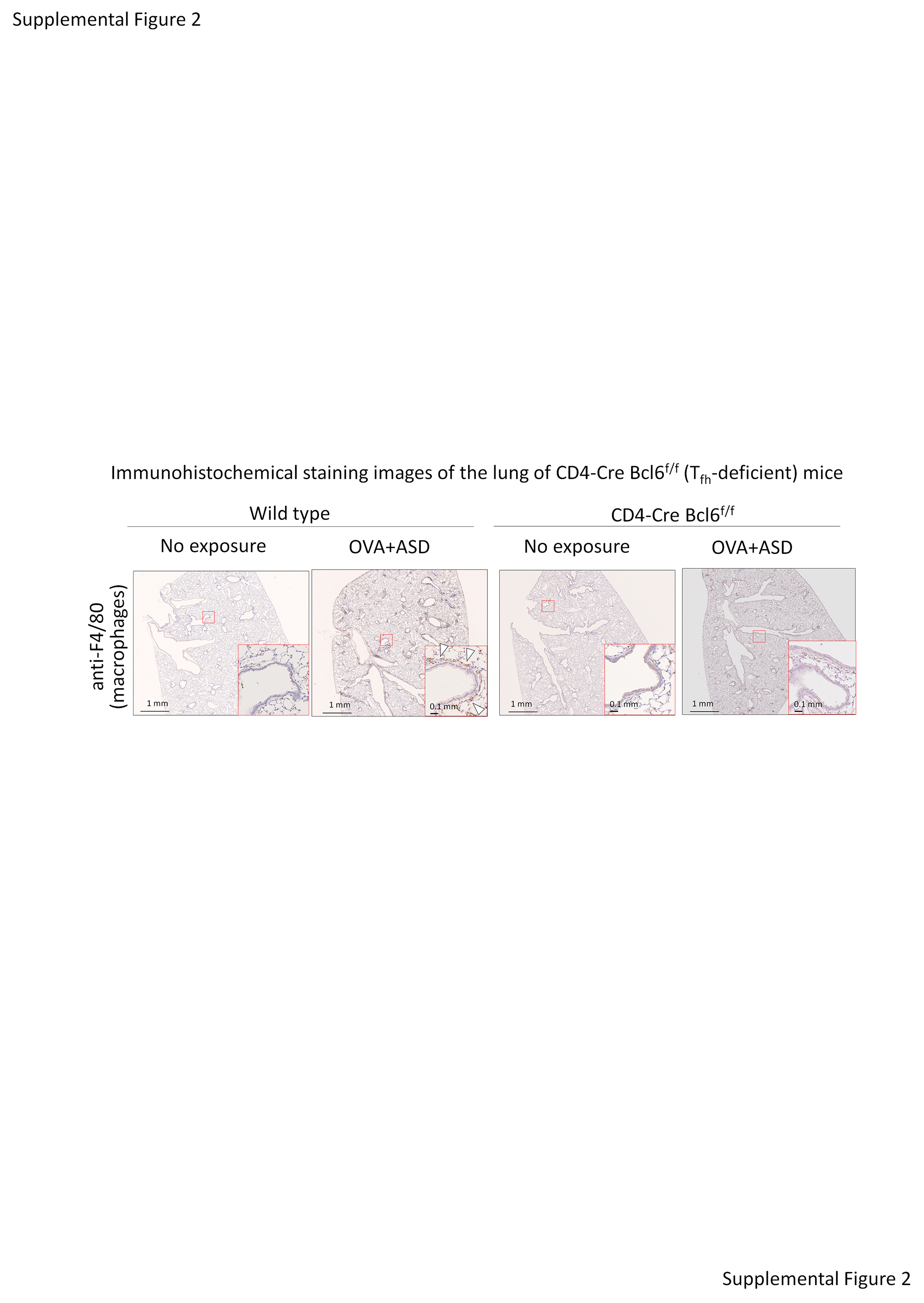

Supplement: Supplementary file 2 [file Image2.tif]
